# Polypyrimidine Tract-Binding Protein Induces p19^Ink4d^ Expression and Inhibits the Proliferation of H1299 Cells

**DOI:** 10.1371/journal.pone.0058227

**Published:** 2013-03-11

**Authors:** Shankung Lin, Ming Jen Wang, Kuo-Yun Tseng

**Affiliations:** 1 Institute of Cellular and System Medicine, National Health Research Institutes, Zhunan Town, Miaoli, Taiwan, Republic of China; 2 Graduate Institute of Basic Medical Science, China Medical University, Taichung, Taiwan, Republic of China; Institut de Génomique Fonctionnelle de Lyon, France

## Abstract

The expression of polypyrimidine tract-binding protein (PTB) is up-regulated in many types of cancer. Here, we studied the role of PTB in the growth of non small cell lung cancer cells. Data showed that PTB overexpression inhibited the growth of H1299 cells at least by inhibiting DNA synthesis. Quantitative real-time PCR and Western blot analyses showed that PTB overexpression in H1299 cells specifically induced the expression of p19^Ink4d^, an inhibitor of cyclin-dependent kinase 4. Repression of p19^Ink4d^ expression partially rescued PTB-caused proliferation inhibition. PTB overexpression also inhibited the growth and induced the expression of p19^Ink4d^ mRNA in A549 cells. However, Western blot analyses failed to detect the presence of p19^Ink4d^ protein in A549 cells. To address how PTB induced p19^Ink4d^ in H1299 cells, we showed that PTB might up-regulate the activity of p19^Ink4d^ gene (*CDKN2D*) promoter. Besides, PTB lacking the RNA recognition motif 3 (RRM3) was less effective in growth inhibition and p19^Ink4d^ induction, suggesting that RNA-binding activity of PTB plays an important role in p19^Ink4d^ induction. However, immunoprecipitation of ribonuclearprotein complexes plus quantitative real-time PCR analyses showed that PTB might not bind p19^Ink4d^ mRNA, suggesting that PTB overexpression might trigger the other RNA-binding protein(s) to bind p19^Ink4d^ mRNA. Subsequently, RNA electrophoretic mobility-shift assays revealed a 300-base segment (designated as B2) within the 3′UTR of p19^Ink4d^ mRNA, with which the cytoplasmic lysates of PTB-overexpressing cells formed more prominent complexes than did control cell lysates. Insertion of B2 into a reporter construct increased the expression of the chimeric luciferase transcripts in transfected PTB-overexpressing cells but not in control cells; conversely, overexpression of B2-containing reporter construct in PTB-overexpressing cells abolished the induction of p19^Ink4d^ mRNA. In sum, we have shown that PTB plays as a negative regulator in H1299 cell proliferation at least by inducing p19^Ink4d^ expression at transcriptional and post-transcriptional levels.

## Introduction

The polypyrimidine-tract binding protein (PTB), also known as heterogeneous nuclear ribonucleoprotein I (hnRNP I), is a 57-kDa RNA-binding protein containing four RNA recognition motifs (RRMs) which bind preferentially to pyrimidine-rich sequences [1]. PTB has been implicated in the synthesis and maturation of mRNA including alternative splicing [2], translocation [3], and polyadenylation [4]. PTB is also involved in internal ribosomal entry site-dependent translation [5], and modulation of mRNA stability [6]–[10]. Therefore, PTB may be involved in physiological activities by regulating global gene expression. Indeed, PTB has been found to participate in a spectrum of processes ranging from embryonic development [11] to pathology of several diseases including cancer.

Expression of PTB is often increased in cancer cells. Several studies have shown the involvement of PTB in regulating cell proliferation and invasion. For examples, PTB knockdown has been shown to reduce the growth of several types of tumor cells [12], [13]. A recent paper reported that PTB is able to switch the expression of pyruvate kinase from M1 to M2 isoform, which may promote aerobic glycolysis to support the rapid proliferation of cancer cells [14]. These findings suggest that PTB may act as a positive regulator in the growth and expansion of cancer cells. However, our previous studies using a metastatic cell model derived from lung adenocarcinoma have shown that (i) PTB can down-regulate the expression of hypoxia-inducible factor 1α which is a critical factor helping tumor cells cope with harsh microenvironment resulted from vigorous proliferation of tumor cells, (ii) PTB expression is down-regulated in cells possessing higher metastatic capability, and (iii) PTB is able to inhibit cell invasion [10]. These findings suggest that PTB might act as a negative regulator in the development of metastatic phenotypes in lung cancer cells, but it was not clear if and how PTB might regulate the growth of lung cancer cells.

In this report, we study the influence of PTB on lung cancer cell growth. We have identified *CDKN2D* as a target gene of PTB in regulating the growth of H1299 cells. *CDKN2D* encodes the p19^Ink4d^, a member of the INK4 family of cyclin-dependent kinase inhibitors (CKIs). For convenience, we used the term ‘p19^Ink4d^ gene’ to represent *CDKN2D*. It is well known that p19^Ink4d^ inhibits CDK4 activity which otherwise interacts with cyclin D1 to promote G1 progression. We have also investigated the molecular mechanism(s) by which PTB induces the expression of p19^Ink4d^ in H1299 cells.

## Materials and Methods

### Cell Culture

H1299 and A549 cells were purchased from American Type Culture Collection (ATCC), and were maintained in RPMI 1640 and F12 medium (GIBCO-BRL), respectively. Culture medium was supplemented with 10% fetal bovine serum (FBS), glutamine, penicillin, and streptomycin. Cells were cultured at 37°C in a humidified atmosphere containing 5% CO_2_.

### Plasmids Construction, Transfection, and Luciferase Assay

To assess if PTB regulated p19^Ink4d^ gene promoter activity, a DNA fragment corresponding to the region from –1278 to +11 of this gene was cloned into the Kpn I-Xho I site of pGL3-basic vector (Promega) to generate pGL3-p19-luc. For PTB overexpression, cells were transfected with pCMV-SPORT6-PTB or empty vector (http://www.bcrc.firdi.org.tw). For PTB knockdown, cells were transfected with siRNA_PTB_ (Qiagen, catalogue no. SI00301490) or siRNA_GFP_ (Dharmacon, catalogue no. D-001300-01-20). To prepare constructs expressing partially deleted forms of PTB, PCRs were carried out using pCMV-SPORT6-PTB as template to generate pPTB-de-1, pPTB-de-2, pPTB-de-3, and pPTB-de-4 constructs harboring PTB cDNA lacking of RRM1, RRM2, RRM3, or RRM4 sequence, respectively [10]. RT-PCR analyses were performed to examine the expression of the intact and partially deleted forms of PTB mRNAs in transfected cells. The 5′ and 3′ primers used were: CCATGGACGGCATTGTCCC and CTAGATGGTGGACTTGGAGAAGGAG. To examine the influence of specific regions of p19^Ink4d^ mRNA on the expression of a luciferase reporter, DNA fragments B2 and C3 were cloned into the Xba I site of pGL3-promoter vector to generate pGL3-B2 and pGL3-C3, respectively. Transfection was conducted using the Effectene reagent (Qiagen). Cells were transfected for 8 h, and then incubated in regular medium for 16 h which was defined as 0 h post-transfection. Luciferase assay was conducted using the Dual-Luciferase Reporter Assay System (Promega).

### Cell Proliferation Assay

Cell proliferation was determined by measuring the incorporation of 5-bromo-2-deoxyuridine (BrdU) into DNA using BrdU Cell Proliferation Assay kit (Millipore, MA, USA), and by the colorimetric 3-[4,5-dimethylthiazol-2-yl]-2,5-diphenyl tetrazolium bromide (MTT) assay. For BrdU incorporation assay, cells were seeded into 96-well microplate and cultured in regular medium for 20 h. Then, either BrdU or PBS was added in medium, and cells were cultured for 4 h. Subsequently, cells were subjected to detection of the BrdU signals. For MTT assay, cells were treated with 0.05% (w/v) MTT (Sigma, MO, USA) for 4 h at 37°C. After the removal of MTT solution, cells were treated with 100 µl DMSO. The optical density was determined using a microplate reader at a wavelength of 540 nm.

### Flow Cytometric Analysis

Cells were trypsinized and fixed with prechilled 80% (v/v) ethanol. After centrifugation, cell pellets were resuspended in 0.5% Triton X-100 for 5 min, and treated with propidium iodide (50 µg/ml) plus 0.5% (w/v) RNase A for 10 min. The DNA content of cell samples was analyzed by the FACS calibur flow cytometer (BD Biosciences) with an argon laser tuned to the 488 nm line for excitation.

### Western Blot Analysis

Forty µg of whole-cell lysate was resolved on SDS-polyacrylamide gels and transferred onto polyvinylidene difluoride membranes, and hybridized with anti-PTB (Abcam), anti-cyclin A1, B1, D1, E1 (Santa Cruz), anti-p16^Ink4a^ (Millipore), anti-p19^Ink4d^ (Genetex), anti-p21^Cip1^ (Millipore), anti-p27^Kip1^ (Millipore), and anti-β-actin (Chemicon) antibodies. Signals were obtained by enhanced chemilluminescence (PIERCE).

### Quantitative Real-time PCR (RT-qPCR) Analysis

Total RNA was isolated from cells using Trizol (Life Technologies) and subjected to reverse transcription-PCRs (RT-PCRs) to generate complimentary DNAs. RT-qPCR was performed using the Power SYBR Green PCR Master Mix (Applied Biosystems, CA). The 5′ and 3′ primers used were as follows: PTB, CCTGCAGGCGGTGAACTCGG and CCATCGCCATCCCTGCGTCC; cyclin A1, TGAACTACATTGATAGGTTCCTGT and TGACTGTTGTGCATGCTGTGGTGC; cyclin D1, GCCAACCTCCTCAACGACCGG and GTCCATGTTCTGCTGGGCCTG; p19^Ink4d^, GTGCATCCCGACGCCCTCAAC and TGGCACCTTGCTTCAGCAGCTC; luciferase, GCACTCTGATTGACAAATACG and CTCGGGTGTAATCAGAATAGC; and β-actin, CCCTGGCACCCAGCAC and GCCGATCCACACGGAGTAC. All RT-qPCRs were performed in triplicate on an ABI PRISM 7000 Sequence Detector System [10]. The relative mRNA levels were calculated using the 2^−ΔΔCT^ method, with β-actin mRNA as a normalizer.

### Immunoprecipitation of Ribonucleoprotein Complexes

To assess the binding of PTB-containing protein complexes on the p19^Ink4d^ mRNA of H1299 cells, cells were processed and the antibody-coated protein A beads were prepared as described [10]. For immunoprecipitation of ribonucleoprotein complexes, the antibody-coated beads were mixed with 1 mg of cell lysate, incubated at 4°C with gentle shaking for 2 h, and then washed as described [10]. RNAs were isolated from the precipitated ribonucleoprotein complexes and subjected to RT-qPCR analyses.

### Preparation of Radiolabeled RNA Transcripts and RNA Electrophoretic Mobility-shift Assays (REMSA)

Total RNA prepared from H1299 cells was used for RT-PCRs to generate various regions of p19^Ink4d^ cDNA. A T7 RNA polymerase promoter sequence (T7) was placed 5′ to the 5′ primers used in this study. The 5′ primers used were as follows: A, (T7)TCTGGGGTCACCCTCTCC; B, (T7)ACGAGACCCAAGGGCAGAG; and C, (T7)GGTGTTGGTTTTGGGGGTGT. The 3′ primers used were as follows: 1, CTCTGCCCTTGGGACTCG; 2, GATCATGCACAAGTCTTAATTTAA; and 3, ACACCCCCAAAACCAACACC. PCR-amplified products were purified to serve as templates for synthesis of radiolabeled RNA probes [10]. REMSA assays were performed as described previously [10].

### Statistical Analysis

Data shown were the mean ± S.D. Statistical difference between two groups was determined by paired t-test. A value of P<0.05 was considered to denote statistical significance.

## Results

### PTB Inhibited the Growth of H1299 Cells at Least by Inhibiting its Proliferation

**Figure 1:**
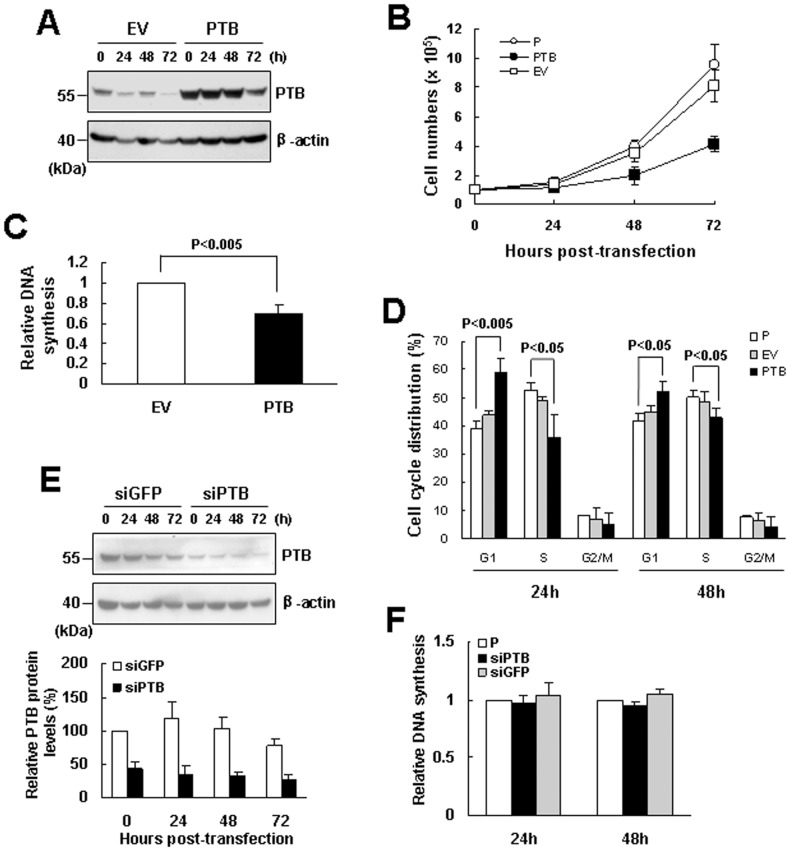
Overexpression of PTB inhibited the proliferation of H1299 cells. (A) Western blot analyses. H1299 cells (1×10^6^) were transiently transfected with either 2 µg pCMV-SPORT6 (EV) or equal molar of pCMV-SPORT6-PTB (PTB) for 8 h, after cultured for 16 h (designated as 0 h post-transfection), cells were harvested every 24 h as indicated. Cell lysates were analyzed for the levels of PTB and β-actin. A representative blot is shown. (B) Cells were either left untreated (P) or transfected with pCMV-SPORT6 (EV) or pCMV-SPORT6-PTB (PTB) and harvested as described above. Cell numbers were determined using a hemacytometer. Values shown are the means ± S.D. from three independent experiments. (C) BrdU incorporation assays. Transfected cells harvested at 0 h post-transfection as described in (A) were seeded into 96-well culture plate (1.2×10^2^ cells/well) and subjected to BrdU incorporation assays. The relative DNA synthesis was calculated by comparing the BrdU signals of cells overexpressing PTB to that of EV control cells (to which a value of 1 was assigned). Data represent the means ± S.D. from three independent analyses. (D) Flow cytometric analyses. Parental cells (P) or transfected cells as described in (A) were harvested 24 and 48 h post-transfection and subjected to flow cytometric analyses for their DNA content. (E) PTB knockdown and BrdU incorporation assays. Cells were either left untreated (P) or transfected with 100 nM of siRNA_PTB_ (siPTB) or siRNA_GFP_ (siGFP, as a control) for 8 h, and cultured for 16 h without transfection reagent. Cells were then harvested every 24 h as indicated. Western blot analyses were performed to show the PTB levels in cells receiving either siPTB or siGFP. A representative blot is shown. PTB signals were quantitated and normalized to corresponding β-actin signals. Relative expression levels were calculated by comparing all the normalized signals to that of control cells of 0 h (to which a value of 100 was assigned). Values shown are the means ± S.D. from three independent experiments. Meanwhile, cells harvested 24 and 48 h post-transfection were seeded into 96-well culture plate (1.2×10^2^ cells/well) and subjected to BrdU incorporation assays (F). The relative DNA synthesis was calculated by comparing the BrdU signals of cells receiving siPTB or siGFP to that of untreated parental cells (to which a value of 1 was assigned). Data represent the means ± S.D. from three independent analyses.

To observe the effect of PTB on cell growth, we overexpressed PTB transiently in H1299 cells. Western blot analyses were performed to show the PTB levels in PTB-overexpressing and corresponding control cells harvested 0, 24, 48, and 72 h post-transfection (Fig. 1A). In parallel, we also counted cell numbers 0, 24, 48, and 72 h post-transfection. The results showed that overexpression of an empty vector slightly decreased cell growth, which however did not reach to statistical significance. Nonetheless, the inhibitory effect of PTB overexpression on cell growth was observed as early as 24 h post-transfection (P<0.05) (Fig. 1B). BrdU incorporation assays performed 24 h post-transfection revealed that the DNA synthetic activity in cells overexpressing PTB was approximately 30% less than that of corresponding control (Fig. 1C). Subsequently, we performed flow cytometric analyses to examine the impact of PTB overexpression on cell cycle progression. As shown, at the time 24 h post-transfection, ∼59% and ∼36% of PTB-overexpressing cells were at G1 and S phases, respectively, whereas those of parental cells were ∼39% and ∼53%, respectively (Fig. 1D). At the time 48 h post-transfection, ∼52% and ∼43% of PTB-overexpressing cells were at G1 and S phases, respectively, whereas those of parental cells were ∼42% and ∼50%, respectively. Overexpression of a control vector did not affect cell cycle progression. These results indicated that PTB could inhibit H1299 cell growth at least by inhibiting the G1-to-S transition of cell cycle. It is worthy to note that ∼0.41% and ∼0.44% of PTB-overexpressing cells were at sub-G1 phase as measured 24 and 48 h post-transfection, while those of corresponding control cells were ∼0.45% and ∼0.38%, respectively. In comparison, we examined if PTB knockdown stimulated DNA synthesis. We overexpressed small interfering RNA (siRNA) targeting either PTB or green fluorescent protein (GFP) mRNA in H1299 cells. Western blot analyses were performed to confirm the knockdown of PTB expression. As shown, the PTB levels in cells receiving siPTB were approximately 43%, 34%, 32%, and 26% of that of the control cells 0, 24, 48, and 72 h post-transfection (Fig. 1E). However, PTB knockdown seemed not to affect DNA synthesis as evidenced by BrdU incorporation assays (Fig. 1F).

### Overexpression of PTB Induced the Expression of p19^Ink4d^ in H1299 Cells

**Figure 2:**
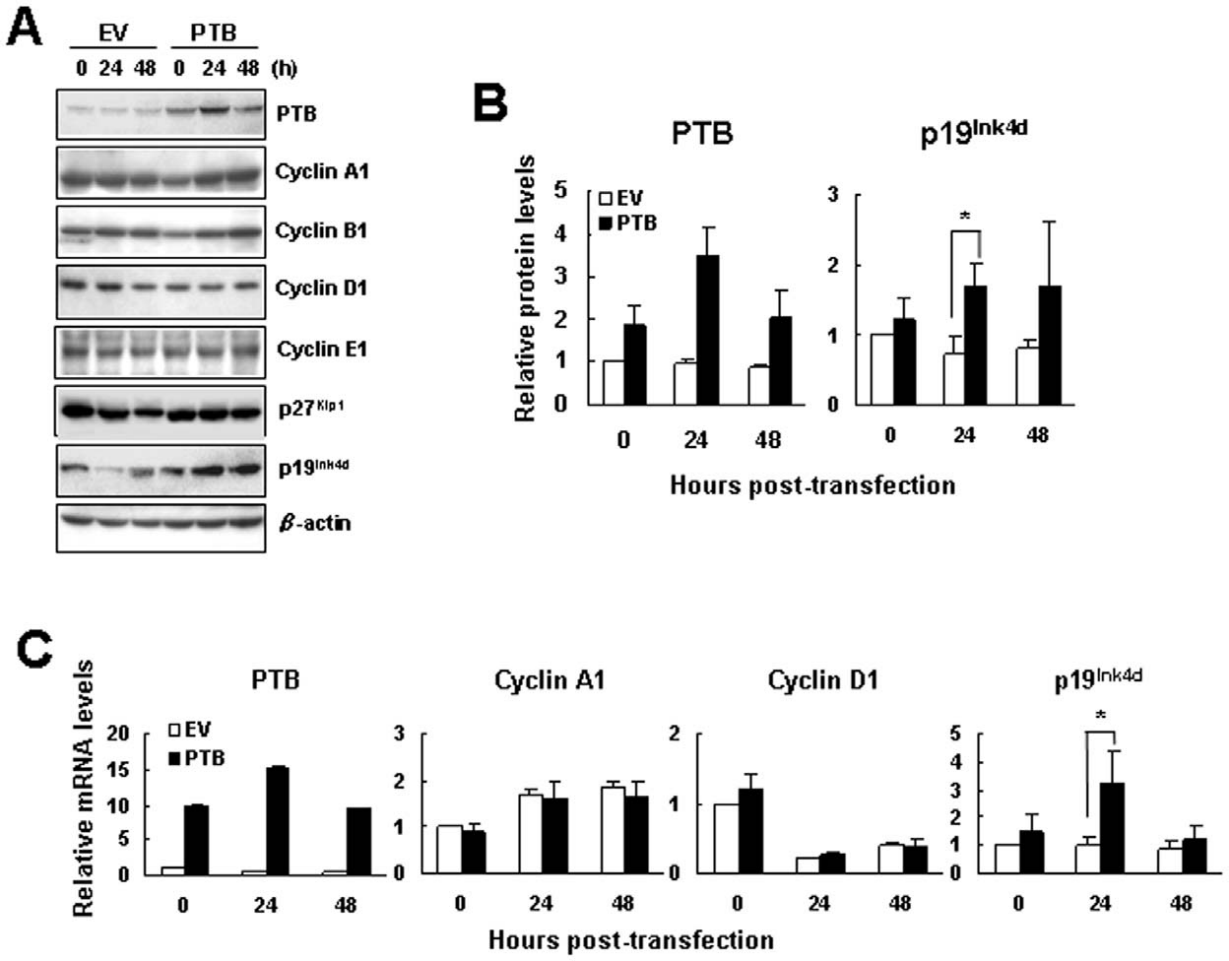
Overexpression of PTB specifically induced the expression of p19^Ink4d^. (A) Western blot analyses. Cells were processed as described in figure 1A, and subjected to Western blot analyses for the levels of cyclins, CKIs and β-actin as indicated. Representative blots are shown. Signals of PTB and p19^Ink4d^ were quantitated and normalized to corresponding β-actin signals. Relative expression levels were calculated by comparing all the normalized signals to that of EV control of 0 h (to which a value of 1 was assigned). Data represent the means ± S.D. from three independent experiments. *P<0.05 versus corresponding control (B). (C) RT-qPCR analyses. RNAs were isolated from cells processed as described in (A), and subjected to RT-qPCR analyses for the levels of PTB, cyclins A1 and D1, p19^Ink4d^, and β-actin mRNAs. Relative expression levels were calculated by comparing all the normalized levels to that of EV control of 0 h (to which a value of 1 was assigned). Data represent the means ± S.D. from three independent experiments. *P<0.05 versus corresponding control.

So then, we examined the influence of PTB overexpression to the expression of several cyclins and CKIs. Western blot analyses showed that PTB overexpression in H1299 cells did not affect the expression of cyclins A1, B1, D1, and E1, and p27^Kip1^, but induced the expression of p19^Ink4d^ (Fig. 2A). As measured 24 h post-transfection, a 3.5-fold increase in PTB expression was associated with a 1.7-fold increase in p19^Ink4d^ expression (Fig. 2B). The other CKIs such as p16^Ink4a^ and p21^Cip1^ were not detected by Western blot analyses in H1299 cells (data not shown). Besides, we also performed quantitative real-time PCR (RT-qPCR) analyses to examine the mRNA levels of PTB, cyclin A1, cyclin D1, and p19^Ink4d^ 0, 24, and 48 h post-transfection. The results showed that PTB overexpression specifically induced the expression of p19^Ink4d^ mRNA 24 h post-transfection (Fig. 2C).

### PTB Overexpression also Inhibited the Growth of A549 Cells

**Figure 3:**
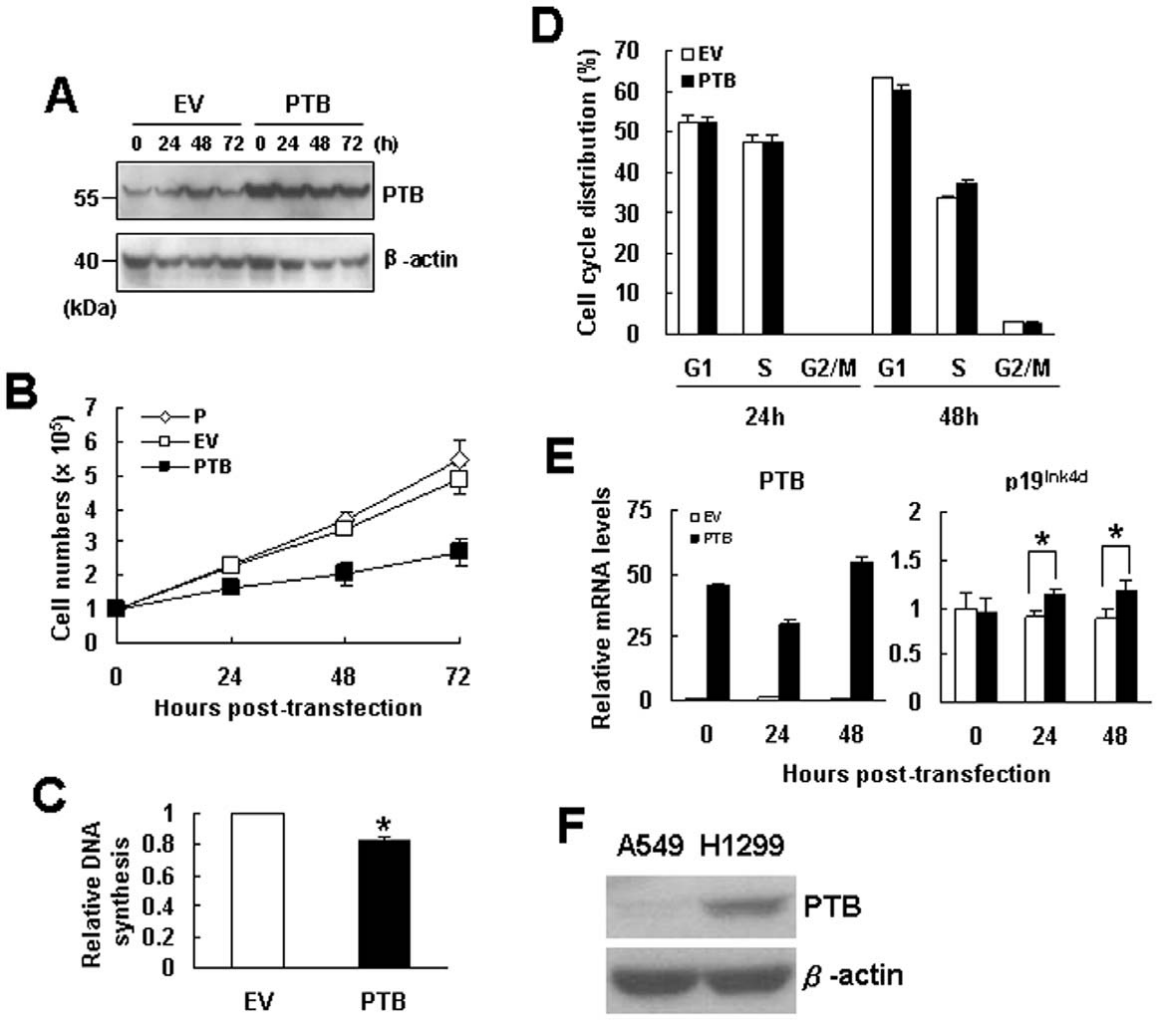
Overexpression of PTB inhibited the proliferation of A549 cells. (A) Western blot analyses. A549 cells (1×10^6^) were transiently transfected with either 2 µg pCMV-SPORT6 (EV) or equal molar of pCMV-SPORT6-PTB (PTB) for 8 h, after cultured for 16 h (designated as 0 h post-transfection), cells were harvested every 24 h as indicated. Cell lysates were analyzed for the levels of PTB and β-actin. A representative blot is shown. (B) Cells were either left untreated (P) or transfected with pCMV-SPORT6 (EV) or pCMV-SPORT6-PTB (PTB) and harvested as described above. Cell numbers were determined using a hemacytometer. Values shown are the means ± S.D. from three independent experiments. (C) BrdU incorporation assays. Transfected cells harvested at 0 h post-transfection as described in (A) were seeded into 96-well culture plate (1.2×10^2^ cells/well) and subjected to BrdU incorporation assays. The relative DNA synthesis was calculated by comparing the BrdU signals of cells overexpressing PTB to that of EV control cells (to which a value of 1 was assigned). Data represent the means ± S.D. from three independent analyses. *P<0.0005 versus corresponding control. (D) Flow cytometric analyses. Transfected cells as described in (A) were harvested 24 and 48 h post-transfection and subjected to flow cytometric analyses for their DNA content. (E) RT-qPCR analyses. RNAs were isolated from cells processed as described in (A), and subjected to RT-qPCR analyses for the levels of PTB, p19^Ink4d^, and β-actin mRNAs. Relative expression levels were calculated by comparing all the normalized levels to that of EV control of 0 h (to which a value of 1 was assigned). Data represent the means ± S.D. from three independent experiments. *P<0.005 versus corresponding control. (F) Western blot analyses. Forty micrograms of lysates prepared from H1299 and A549 cells were analyzed for their cellular levels of p19^Ink4d^ protein. A representative blot is shown.

We also examined if PTB overexpression inhibits the growth of another non small cell lung cancer cell line A549. We overexpressed PTB transiently in A549 cells and performed Western blot analyses to check PTB levels in cells harvested 0, 24, 48, and 72 h post-transfection (Fig. 3A). By counting the cell numbers, we found that PTB overexpression also inhibited cell growth (Fig. 3B). BrdU incorporation assays performed 24 h post-transfection revealed that the DNA synthetic activity in cells overexpressing PTB was approximately 17% less than that of corresponding control (Fig. 3C). These results indicated that PTB inhibited the growth of A549 cell at least by inhibiting its proliferation. Subsequently, we performed flow cytometric analyses to examine the impact of PTB overexpression on cell cycle progression. The data showed that PTB overexpression did not block G1-to-S transition of the cell cycle (Fig. 3D). Even so, we examined if PTB overexpression also induced p19^Ink4d^ expression in A549 cells. We performed RT-qPCR analyses to examine the mRNA levels of PTB and p19^Ink4d^ in cells harvested 0, 24, and 48 h post-transfection. The results showed that PTB overexpression induced approximately 1.1- and 1.2-fold increase in p19^Ink4d^ expression (Fig. 3E). However, Western blot analyses did not detect p19^Ink4d^ in A549 cells with or without PTB overexpression (data not shown). Because of this, we also examined cellular levels of PTB protein in H1299 and A549 cells, and found that H1299 cells expressed more PTB than A549 cells (Fig. 3F).

### Knockdown of p19^Ink4d^ Partially Reversed PTB-induced Inhibition of H1299 Cell Proliferation

**Figure 4:**
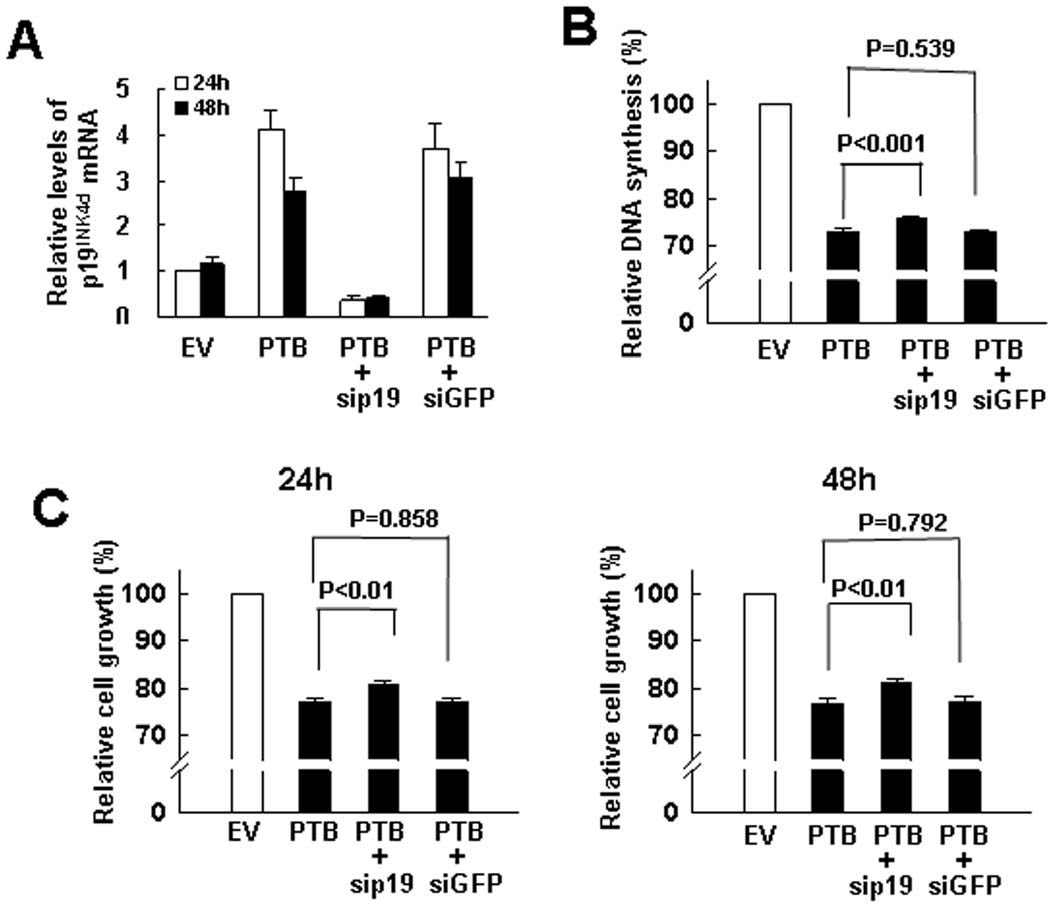
Repression of p19^Ink4d^ expression partially abolished PTB’s inhibitory effect on cell proliferation. H1299 cells (1×10^6^) were transfected with either 2 µg pCMV-SPORT6 (EV) or equal molar of pCMV-SPORT6-PTB (PTB), or pCMV-SPORT6-PTB plus 75 nM siRNA_p19_^Ink4d^ (sip19), or pCMV-SPORT6-PTB plus 75 nM siRNA_GFP_ (siGFP), cells were harvested 24 and 48 h post-transfection. (A) RT-qPCR analyses. Cells harvested 24 and 48 h post-transfection were measured for the levels of PTB mRNA and normalized to β-actin signals. Relative expression levels were calculated by comparing all the normalized signals to that of EV control of 24 h (to which a value of 1 was assigned). Data represent the means ± S.D. from three independent experiments. (B) BrdU incorporation assays. Cells harvested 0 h post-transfection were seeded into 96-well microplate for BrdU incorporation assays. The relative DNA synthesis was calculated by comparing the BrdU signals of PTB-overexpressing cells to that of EV control (to which a value of 100 was assigned). Data represent the means ± S.D. from three independent analyses. (C) MTT assays. Cells harvested 0 h post-transfection were seeded into 96-well microplate (1200 cells/well), cultured in regular medium for 20 (left) and 44 h (right), and subjected to MTT assays in the next 4 h (see Materials and Methods). Data represent the means ± S.D. from three independent analyses.

Since PTB overexpression in H1299 cells caused G1-arrest, and p19^Ink4d^ is a CDK4 inhibitor, our data suggested that p19^Ink4d^ induction might play a role in PTB’s inhibitory effect on H1299 cell proliferation. Using small interfering RNA (siRNA) targeting p19^Ink4d^ mRNA for degradation, we were able to knock down PTB-induced p19^Ink4d^ expression in H1299 cells 24 and 48 h post-transfection (Fig. 4A). Subsequent BrdU incorporation assays performed 24 h post-transfection showed that while PTB overexpression caused ∼27% decrease in DNA synthesis, concomitant inhibition of p19^Ink4d^ expression recovered DNA synthesis by ∼3% (Fig. 4B). Moreover, MTT assays showed that while PTB caused ∼23% and ∼24% decrease in cell proliferation 24 and 48 h post-transfection, respectively, concomitant inhibition of p19^Ink4d^ expression also recovered cell proliferation by ∼3.8% and ∼4.3%, respectively (Fig. 4C). Taken together, these data indicated that p19^Ink4d^-induction was involved in PTB’s inhibitory effect on H1299 cell proliferation.

### PTB Overexpression Activated the Promoter Activity of p19^Ink4d^ and Cyclin D1 Genes

**Figure 5:**
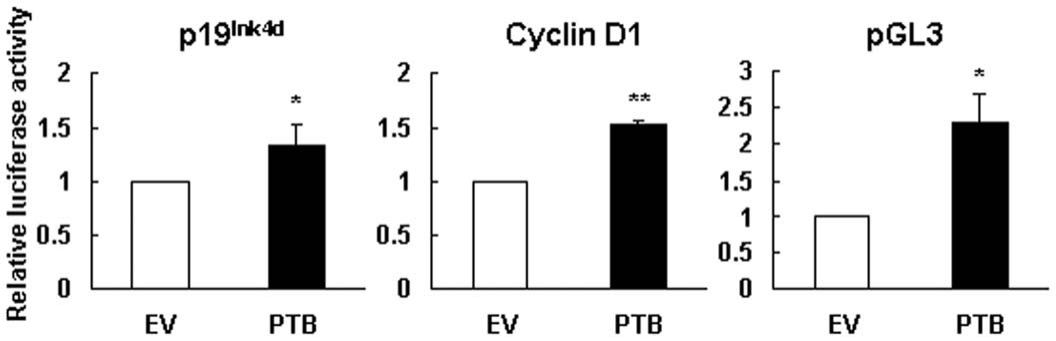
Overexpression of PTB activated the p19^Ink4d^ and cyclin D1 genes promoters. H1299 cells (1×10^6^) were transiently transfected with either 2 µg pCMV-SPORT6 (EV) or equal molar of pCMV-SPORT6-PTB (PTB). At the time 0 h post-transfection, transfected cells were transfected with either 2 µg pGL3-p19-luc or equal molar of a plasmid harboring a luciferase reporter gene driven by cyclin D1 or SV40 promoter (pGL3). Cotransfection with a Renilla luciferase reporter (0.1 µg) served as an internal control for normalization of transient transfection. After incubation in regular medium for 16 h, cells were harvested for luciferase assays. Relative luciferase activity was calculated by comparing all the normalized signals to that of EV control (to which a value of 1 was assigned). Data represent the means ± S.D. from three independent analyses. *P<0.05; **P<0.01 versus EV control.

Next, we investigated how PTB induces the expression of p19^Ink4d^ mRNA. We first examined if PTB activated p19^Ink4d^ gene promoter in H1299 cells. PTB-overexpressing and control cells were transfected with a plasmid harboring a luciferase reporter under control of the p19^Ink4d^ gene promoter. Subsequent luciferase assays showed that the luciferase activity of PTB-overexpressing cells was approximately 1.3 folds of that of control cells (Fig. 5). Since PTB overexpression did not induce cyclin D1 expression (Fig. 2C), we also transfected PTB-overexpressing and control cells with a plasmid harboring a luciferase reporter under control of the cyclin D1 promoter. Interestingly, luciferase assays showed that the luciferase activity of PTB-overexpressing cells was approximately 1.5 folds of that of control cells. Next, we transfected PTB-overexpressing and control cells with a luciferase reporter driven by SV40 promoter (pGL3-promoter), and found that the luciferase activity of PTB-overexpressing cells was approximately 2.3 folds of that of control cells.

### RNA-binding Activity of PTB was Important for Proliferation Inhibition and p19^Ink4d^ Induction

**Figure 6:**
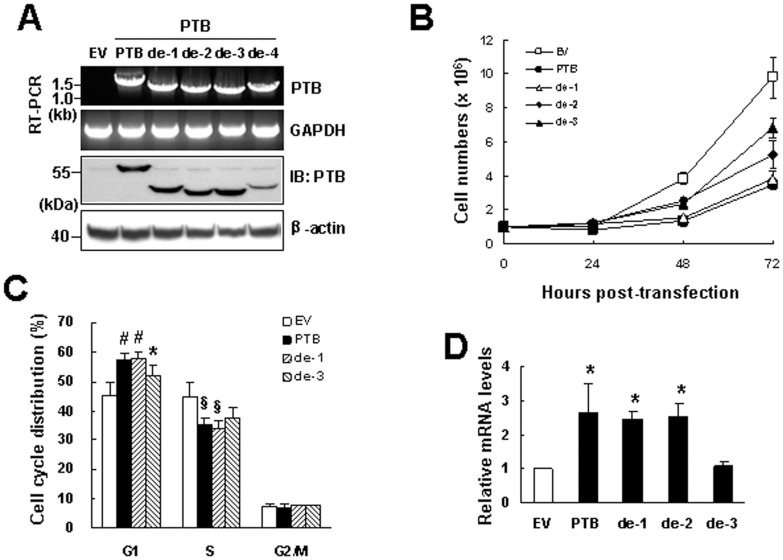
The effect of partially deleted forms of PTB to the growth of H1299 cells. H1299 cells (1×10^6^) were transfected with either 2 µg pCMV-SPORT6 (EV) or equal molar of pCMV-SPORT6-PTB (PTB), pPTB-de-1 (de-1), pPTB-de-2 (de-2), pPTB-de-3 (de-3) or pPTB-de-4 (de-4). (A) RT-PCR and Western blot analyses. Transfected cells were harvested 24 h post-transfection, and analyzed for the expression of full-length and partially deleted forms of PTB. (B) Transfected cells harvested 0, 24, 48, and 72 h post-transfection were counted using a hemacytometer. Values shown are the means ± S.D. from three independent experiments. (C) Flow cytometric analyses. Transfected cells were harvested 24 h post-transfection, and subjected to flow cytometric analyses for their DNA content. *P<0.05; ^#^P<0.01 versus EV control of G1 phase. §P<0.05 versus EV control of S phase. (D) RT-qPCR analyses. Transfected cells were harvested 24 h post-transfection, and subjected to RT-qPCR analyses for the levels of p19^Ink4d^ and β-actin mRNA. Relative mRNA levels were calculated by comparing all the normalized p19^Ink4d^ signals to that of EV control (to which a value of 1 was assigned). Data represent the means ± S.D. from three independent experiments. *P<0.0001 versus EV control.

On the other hand, we examined if post-transcriptional mechanism(s) is involved in PTB-induced expression of p19^Ink4d^ mRNA. We overexpressed in H1299 cells either full length PTB or truncated PTBs of which one of the RRMs was deleted respectively (designated as de-1, de-2, de-3, and de-4), and examined the involvement of PTB’s RRMs in cell growth regulation. RT-PCR and Western blot analyses confirmed the overexpression of these proteins (Fig. 6A). For unknown reasons, expression of de-4 protein was less than the other forms of PTB. Therefore, de-4 form of PTB was excluded from subsequent experiments. As shown, de-1 was as potent as full length PTB in inhibiting cell growth, whereas de-2 and de-3 exhibited decreasing potency in inhibiting cell growth (Fig. 6B). Subsequently, we examined the effect of full length PTB, de-1, and de-3 on cell cycle progression. Flow cytometric analyses performed 24 h post-transfection showed the capability of these three forms of PTB in arresting cells in the G1 phase (Fig. 6C). However, it seemed that de-3 was less potent than full length PTB although the difference in potency did not reach to statistical significance (P = 0.298). Next, we performed RT-qPCR analyses to examine the potency of de-1, de-2, and de-3 in inducing p19^Ink4d^ mRNA. As shown, full length PTB, de-1, and de-2 exhibited similar potency in p19^Ink4d^ induction, whereas de-3 failed to do so in comparison with corresponding control (Fig. 6D). Taken together, these results indicated that RNA-binding activity played an important role in PTB’s inhibitory effect on H1299 cell proliferation and p19^Ink4d^ induction.

### Identification of PTB-inducible Protein-binding Activities on the 3′-untranslated Region of p19^Ink4d^ mRNA

**Figure 7:**
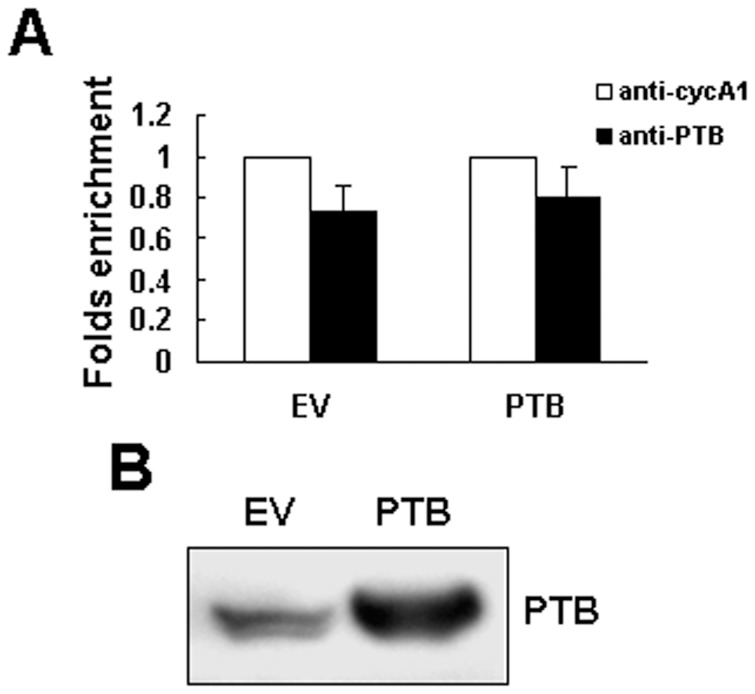
Examination of the binding of PTB-containing protein complexes on p19^Ink4d^ mRNA. (A) Ribonucleoprotein immunoprecipitation and RT-qPCR assays. H1299 cells were transfected with either pCMV-SPORT6 (EV) or pCMV-SPORT6-PTB (PTB), and lysed 24 h post-trsnsfection. One milligram of lysates prepared from EV or PTB cells were incubated with protein A beads precoated with 15 µg of either anti-PTB or anti-cyclin A1 (anti-cycA1) antibody to precipitate ribonucleoprotein complexes and to extract RNAs from the complexes as described in Materials and Methods. RNAs were used in subsequent RT-qPCR assays for p19^Ink4d^ and β-actin mRNAs. p19^Ink4d^ signals were normalized to β-actin signals. The normalized p19^Ink4d^ signals obtained from the ribonucleoprotein complexes pulled down by anti-PTB antibody were compared with those pulled down by anti-cyclin A1 antibody (to which a value of 1 was assigned). Data represent the means ± S.D. from three independent analyses. (B) Western blot analyses. Forty micrograms of the immunoprecipitates pulled down by anti-PTB antibody-coated beads were subjected to Western blot analyses for the levels of PTB. A representative blot is shown.

**Figure 8:**
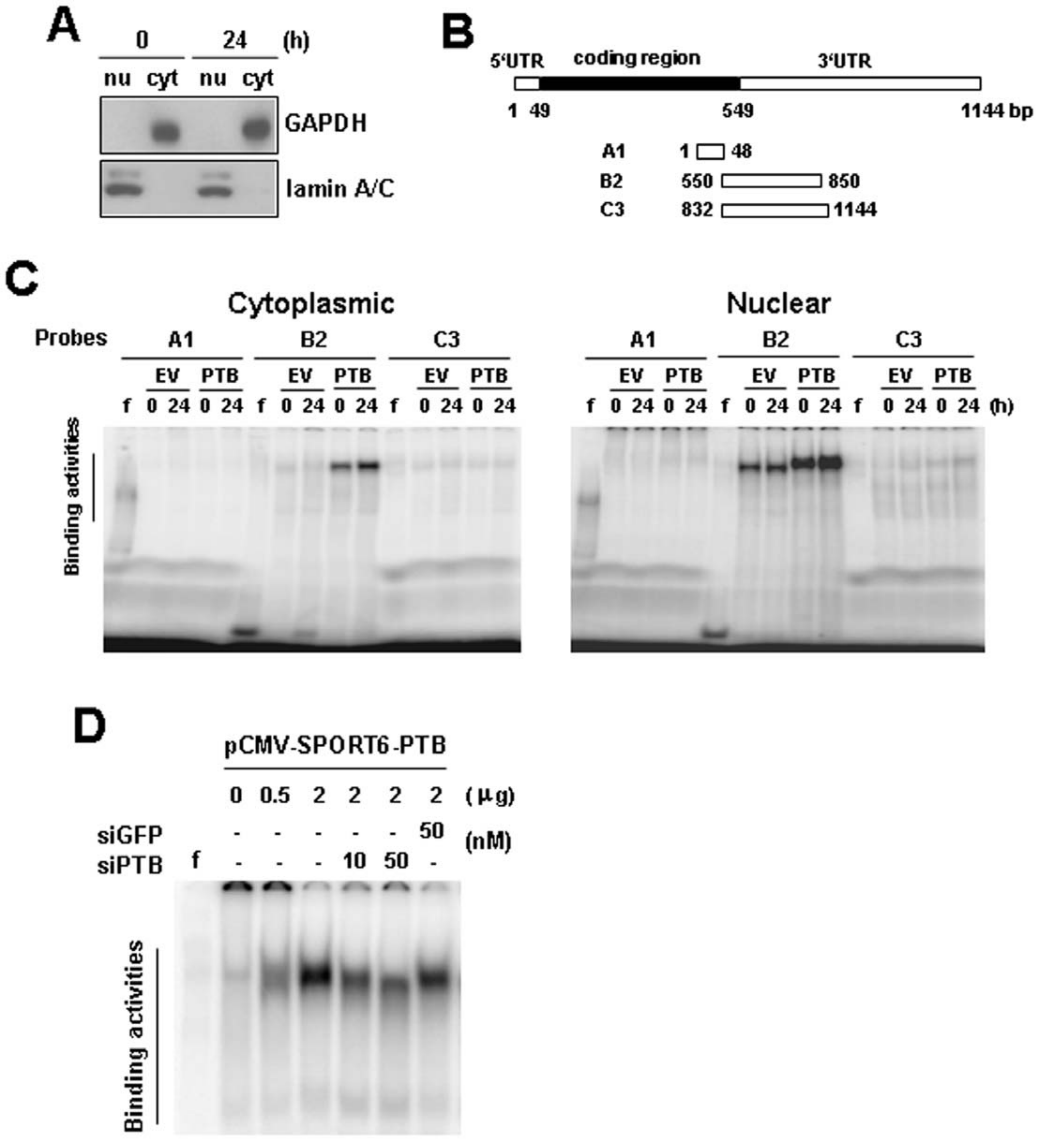
Interactions of cellular proteins and p19^Ink4d^ transcripts. H1299 cells were transfected with either pCMV-SPORT6 (EV) or pCMV-SPORT6-PTB (PTB) as described in figure 1A. Transfected cells harvested 0 and 24 h post-transfection were fractionated into cytoplasmic and nuclear fractions. (A) Western blot analyses. Ten micrograms of nuclear and cytoplasmic fractions prepared from cells receiving pCMV-SPORT6-PTB were analyzed for the presence of GAPDH and lamin A/C. A representative blot is shown. (B) Schematic representation of the p19^Ink4d^ cDNA and various transcripts derived from the 5′UTR and 3′UTR. These transcripts were assayed for protein binding. (C) REMSA assays. Ten micrograms of cytoplasmic or nuclear fraction was mixed with ^32^P-radiolabeled A1, or B2, or C3 probe, and then with RNase T1. Reaction mixtures were resolved on 6% native polyacrylamide gels. Signals were visualized using FLA-2000 (FUJIFILM). f, radiolabeled transcripts digested with RNase T1 without incubation with cell lysate. (D) REMSA assays. Cytoplasmic lysates prepared from H1299 cells transfected with 0, 0.5, or 2 µg of pCMV-SPORT6-PTB, and from cells transfected with 2 µg of pCMV-SPORT6-PTB plus either 10 and 50 nM of siRNA_PTB_ (siPTB) or 50 nM of siRNA_GFP_ (siGFP) were subjected to REMSA assays as described in (C). f, radiolabeled transcripts digested with RNase T1 without incubation with cell lysate.

Accordingly, we performed immunoprecipitation experiments using anti-PTB or control antibody to pull down the ribonucleoprotein complexes in equal amounts of lysates prepared from PTB-overexpressing and control H1299 cells. RNAs were extracted from these immunoprecipitates, and subjected to RT-qPCR analyses for p19^Ink4d^ and β-actin mRNAs. In the lysates of PTB-overexpressing and control H1299 cells, RT-qPCR assays revealed an approximately 0.8-fold and 0.74-fold enrichment in p19^Ink4d^ mRNA in the immunoprecipitate using anti-PTB antibody compared with that in the immunoprecipitate using a control antibody (Fig. 7A). Western blot analysis showed that the immunoprecipitate of PTB-overexpressing cells contained more PTB than that of control cells (Fig. 7B). These data suggested that the PTB-containing protein complexes might not bind p19^Ink4d^ mRNA. Next, we performed RNA EMSA (REMSA) analyses to examine the interactions of p19^Ink4d^ mRNA with cellular proteins prepared from PTB-overexpressing and control H1299 cells harvested at 0 and 24 h post-transfection. These cells were fractionated into cytoplasmic and nuclear fractions. We performed Western blot analyses to examine the purity of the preparations from PTB-overexpressing cells. As shown, the cytoplasmic fractions contained GAPDH but not lamin A/C, whereas the nuclear fractions contained lamin A/C but not GAPDH (Fig. 8A). Subsequently, the cytoplasmic and nuclear fractions were incubated with radiolabeled RNA transcripts corresponding to the 5′UTR (A1) and 3′UTR (B2, C3) of p19^Ink4d^ mRNA (Fig. 8B). As shown, nuclear lysates of PTB-overexpressing cells harvested 0 and 24 h post-transfection formed prominent complexes with B2 transcript with almost identical intensity, and so did that of control cells (Fig. 8C). Cytoplasmic lysates of PTB-overexpressing cells, but not control cells, also exhibited prominent binding activities with B2 transcript. In addition, cytoplasmic lysates of PTB-overexpressing cells harvested 24 h post-transfection formed more complexes with B2 transcript than the lysate prepared from cells harvested 0 h post-transfection (Fig. 8C). Next, we performed REMSA analyses to check the protein-B2 binding activities of cells transfected with increasing amount (0, 0.5, and 2 µg) of PTB-expressing vector. The results showed that the PTB-induced protein-B2 binding activities increased in a dose-dependent manner (Fig. 8D). Moreover, we found that the protein-B2 binding activities were dose-dependently decreased by 10 and 50 nM of siRNA targeting PTB mRNA for degradation (Fig. 8D). These results addressed the specific role of PTB in inducing the protein-B2 binding activities.

### Influence of B2 on the Expression of Chimeric Transcripts

**Figure 9:**
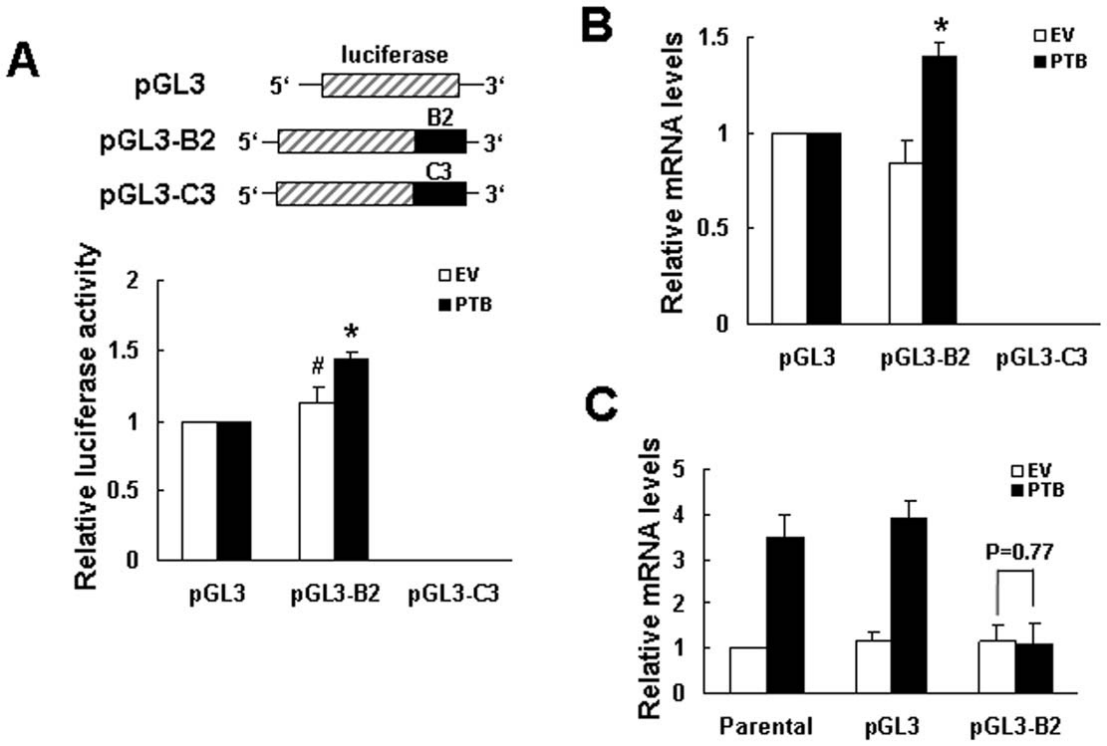
Influence of B2 on the expression of chimeric transcripts and p19^Ink4d^ mRNA. (A) Luciferase assays. H1299 cells were transfected with either pCMV-SPORT6 (EV) or pCMV-SPORT6-PTB (PTB) as described in figure 1A. At the time 24 h post-transfection, transfected cells (1×10^6^) were transfected with either 2 µg of pGL3-promoter (pGL3), or equal molar of pGL3-B2 or pGL3-C3 plasmid together with a Renilla luciferase reporter (0.1 µg). Cells were lysed 24 h post-transfection and subjected to luciferase assays. The luciferase activities of EV and PTB cells receiving pGL3-B2 or pGL3-C3 were compared with that of cells receiving pGL3-promoter (to which a value of 1 was assigned). Data represent the mean ± S.D. from three independent experiments. *P<0.001; ^#^P = 0.181 versus controls. (B) RT-qPCR analyses. Cells processed as described in (A) were analyzed for the levels of luciferase, chimeric luciferase, and β-actin mRNAs. The normalized levels of chimeric luciferase mRNA in cells receiving pGL3-B2 and pGL3-C3 were compared with the normalized levels of luciferase mRNA in cells receiving pGL3 (to which a value of 1 was assigned). Data represent the mean ± S.D. from three independent analyses. *P<0.01 versus cells receiving pGL3. (C) RT-qPCR analyses. Cells were transfected with either pCMV-SPORT6 (EV) or pCMV-SPORT6-PTB (PTB) only (Parental), or transfected with either EV or PTB with cotransfection of either 2 µg of pGL3-promoter (pGL3) or equal molar of pGL3-B2 plasmid together with a Renilla luciferase reporter (0.1 µg). Cells harvested 24 h post-transfection were analyzed for the levels of p19^Ink4d^ and β-actin mRNAs. The normalized levels of p19^Ink4d^ mRNA in cells receiving pGL3 and pGL3-B2 were compared with that of parental EV control (to which a value of 1 was assigned). Data represent the mean ± S.D. from three independent analyses.

Subsequently, we examined the function of B2 in regulating gene expression. PTB-overexpressing and control cells were transiently transfected with pGL3-promoter, or pGL3-B2, or pGL3-C3 plasmid (see Materials and Methods) together with a Renilla luciferase reporter construct (as an internal control) and subjected to luciferase analyses. As shown, compared with the luciferase activity of PTB-overexpressing cells receiving pGL3-promoter, the luciferase activity of PTB-overexpressing cells receiving pGL3-B2 was approximately 44% higher, whereas the luciferase activity of control cells receiving pGL3-B2 was slightly higher than that of control cells receiving pGL3-promoter, but the increase did not reach statistical significance (Fig. 9A). Interestingly, C3 insertion totally abolished luciferase activity in PTB-overexpressing and control cells. Subsequent RT-qPCR analyses showed that the level of the chimeric luciferase transcript in PTB-overexpressing cells receiving pGL3-B2 was approximately 40% higher than the level of luciferase transcript in PTB-overexpressing cells receiving pGL3-promoter, whereas the chimeric luciferase transcript was not detected in cells receiving pGL3-C3 (Fig. 9B). These data suggested that binding of cytoplasmic proteins to the B2 or C3 region might therefore increase or decrease the expression of p19^Ink4d^ mRNA, respectively, in H1299 cells. So, we also examined if overexpression of pGL3 or pGL3-B2 affected the induction of p19^Ink4d^ mRNA by PTB overexpression. RT-qPCR analyses showed that PTB overexpression was able to induce the expression of p19^Ink4d^ mRNA in parental cells and in cells receiving pGL3, whereas overexpression of pGL3-B2 prevented PTB overexpression from inducing p19^Ink4d^ mRNA (Fig. 9C).

## Discussion

In the present study, we investigated the impact of elevated PTB expression to the growth of non small cell lung cancer cells. We have found that PTB overexpression reduces the growth of H1299 and A549 cells at least by inhibiting proliferation. Besides, we found that PTB overexpression also inhibited the growth of CL1-5 lung adenocarcinoma cells as well as the in vitro invasion of H1299 cells (data not shown). Our previous findings have shown the inhibitory effect of PTB overexpression on the in vitro invasion of CL1-5 cells [10]. Thus, although PTB expression is up-regulated in many types of cancers, our results indicate that elevated expression of PTB may not favor the growth and invasion of non small cell lung cancer, which is supported by previous findings that PTB expression is down-regulated in lung adenocarcinoma cells possessing higher invasive and metastatic capability [10]. An unexpected finding is that PTB knockdown did not affect DNA synthesis (Fig. 1F). Our findings are in contrary to the others generated from the studies of ovarian, cervical, and prostate cancers which show that PTB acts as a pro in cell proliferation [12], [13]. It is possible that PTB might regulate tumor growth and invasion in a tissue-dependent manner. However, it is worthy to note that their results were generated by PTB knockdown approach. Based on our data, overexpression of PTB in these three types of cancer cells might provide more complete information to address the role of PTB in regulating the proliferation of cancer cells.

We have also investigated how PTB overexpression down-regulates the proliferation of H1299 and A549 cells. Based on the data that PTB overexpression inhibited DNA synthesis and blocked G1-to-S transition of the cell cycle in H1299 cells (Fig. 1D), we have identified p19^Ink4d^ as a regulatory target of PTB; PTB overexpression induced the expression of p19^Ink4d^ but not the other CDKIs (Fig. 2). Concomitant repression of p19^Ink4d^ expression reversed approximately 11% of the PTB-caused decrease in DNA synthesis and cell growth (Fig. 4), indicating that PTB overexpression might affect the expression of many effecter genes which collectively regulate DNA synthesis, and p19^Ink4d^ gene is one of them. On the other hand, although PTB overexpression also inhibited the proliferation of A549 cells, it seemed not to affect G1-to-S transition of the cell cycle (Fig. 3D). Further investigation showed that PTB overexpression also induced the expression of p19^Ink4d^ mRNA in A549 cells (Fig. 3E). However, Western blot analyses were unable to detect PTB signals in the control and PTB-overexpressing A549 cells (data not shown). Interestingly, A549 cells expressed much less PTB protein than H1299 cells did (Fig. 3F). With these in mind, our data suggest that PTB overexpression might inhibit the proliferation of H1299 and A549 cells via different mechanisms, and that the role of p19^Ink4d^ plays in such a difference requires further investigation.

So then, we examined why PTB overexpression is able to induce p19^Ink4d^ expression in H1299 cells. The fact that PTB overexpression transactivated p19^Ink4d^ promoter suggests that PTB might induce p19^Ink4d^ expression at transcriptional level. However, PTB also activated SV40 promoter and cyclin D1 promoter even though it did not increase the expression of cyclin D1 mRNA (Fig. 5). On one hand, these data imply that transactivation of p19^Ink4d^ gene promoter by PTB overexpression might result from global transcriptional activation given that PTB participates in RNA synthesis. On the other hand, these data imply that post-transcriptional regulation might play a decisive role in presenting the ultimate effects of PTB overexpression on the expression of mRNAs such as p19^Ink4d^ and cyclin D1 in H1299 cells. In consistent with this, we have shown that PTB lacking of RRM3, a domain responsible for RNA-binding, failed to induce p19^Ink4d^ mRNA (Fig. 6D). In addition, we have identified from the 3′UTR of p19^Ink4d^ mRNA a 300-base segment (B2) which while inserted 3′ directly to a luciferase gene could increase the expression of the B2-containing chimeric mRNAs in PTB-overexpressing H1299 cells (Fig. 9B). Furthermore, overexpression of a B2-containing chimeric transcript in PTB-overexpressing H1299 cells, which presumably diluted the protein-binding activities on the B2 region of endogenous p19^Ink4d^ mRNA, abolished the induction of p19^Ink4d^ mRNA by PTB overexpression (Fig. 9C). Taken together, these data support the notion that a post-transcriptional mechanism involving the enhanced interactions between B2 and its binding proteins plays an important role in mediating the induction of p19^Ink4d^ mRNA by PTB overexpression, presumably by increasing the stability of p19^Ink4d^ mRNA in H1299 cells. Although the B2-binding protein complexes are currently uncharacterized, our data suggest that these complexes might not contain PTB (Fig. 7). Interestingly, insertion of C3 3′ directly to a luciferase gene repressed the expression of the C3-containing chimeric mRNAs (Fig. 9B), suggesting a putative post-transcriptional mechanism which keeps the expression of p19^Ink4d^ mRNA down in H1299 cells.

In sum, we have addressed the inhibitory role of PTB in the growth of non small cell lung cancer cells, and revealed a part of the underlying mechanisms. Further comparison of gene expression profiles of PTB-overexpressing and corresponding control cells is expected to reveal more detailed network which links PTB to cell growth inhibition. Our data support the notion that down-regulation of PTB expression might play an important role in the progression of non small cell lung cancer. So then, elucidation of the mechanisms governing the down-regulation of PTB expression is expected to provide critical insight into the development of malignant traits in non small cell lung cancer. In turn, this information could lead to improved treatment strategies.
